# Rare case of pregnancy with vaginal adenocarcinoma

**DOI:** 10.1016/j.ijscr.2020.05.096

**Published:** 2020-06-11

**Authors:** Youjiang Xu, Congqing Li, Bing Wei, Wenyan Wang

**Affiliations:** Department of Obstetrics and Gynecology, The Second Hospital of Anhui Medical University, No. 678 Furong Road, Hefei, Anhui 230601, China

**Keywords:** Pregnancy, Vaginal tumour, Adenocarcinoma

## Abstract

**Introduction:**

Primary vaginal cancer accounts for only 3% of female reproductive tract malignant tumours, and 5% of primary vaginal cancers are adenocarcinomas. Reports of pregnancy with vaginal adenocarcinoma are very rare. There is no guidance for its diagnosis and treatment.

**Case presentation:**

A 27-year-old pregnant woman who had found a vaginal tumour due to vaginal bleeding during pregnancy was diagnosed with vaginal adenocarcinoma by biopsy. The patient chose to wait to treat the cancer until after delivery at full term of pregnancy and then underwent simple vaginal tumour resection and chemotherapy.

**Discussion and conclusion:**

For pregnant women with vaginal adenocarcinoma, a comprehensive assessment and an individualized treatment are needed.

## Highlights

• Primary vaginal cancer accounts for only 3% of the female reproductive tract malignant tumors, of which primary vaginal adenocarcinoma accounts for 5%.
• There is no guidance for its diagnosis and treatment.
• For pregnant women with vaginal adenocarcinoma, comprehensive assessment and individualized treatment are needed.

## 1 Introduction

Primary vaginal cancer accounts for 3% of female reproductive tract malignancies, most of which are squamous cell carcinomas, followed by adenocarcinoma, sarcoma, and melanoma [1]. Because of the rarity of pregnancy with vaginal adenocarcinoma, there are no clinical guidelines to guide treatment.

Here, we present a patient who was diagnosed with vaginal adenocarcinoma during pregnancy and was treated successfully.

This work has been reported in line with the Surgical CAse REport (SCARE) criteria (Agha) [2].

## 2 Case presentation

A 27-year-old patient was admitted to the hospital on February 13, 2019, due to abnormal vaginal bleeding for two months. Her last menstrual period was May 11, 2018. No obvious abnormality was found in the normal prenatal examination at the local hospital. In the middle of December 2018, the patient had abnormal vaginal bleeding at the 29th week of gestation. According to the gynaecological examination without special treatment, there was a new growth on the vaginal wall that was approximately 4 cm × 1 cm × 1 cm. At the 30th week of gestation, the patient had an upper respiratory tract infection. She was admitted to the local hospital and treated with anti-infection treatment. At the same time, she had vaginal bleeding. A new biopsy of the vagina was performed. The biopsy showed that clear cell carcinoma of the vagina was possible. She was recommended to another hospital and underwent a vaginal neoplasm biopsy again. The pathological result was vaginal adenocarcinoma. On February 2, 2019, she was 37 + 5 weeks pregnant and underwent caesarean section at the local hospital. A healthy baby girl weighing 3250 g was born. At that point, the patient still had vaginal bleeding, double breast lactation, no fever and no abdominal pain. The admission diagnosis was vaginal adenocarcinoma.

**Fig. 1:**
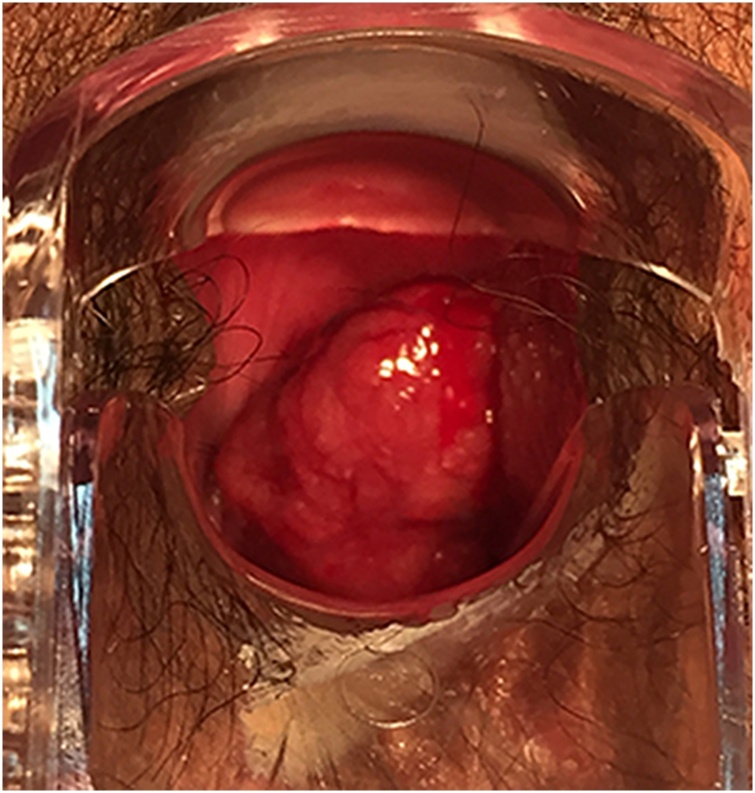
Seeing a vaginal tumor directly (white arrow).

**Fig. 2:**
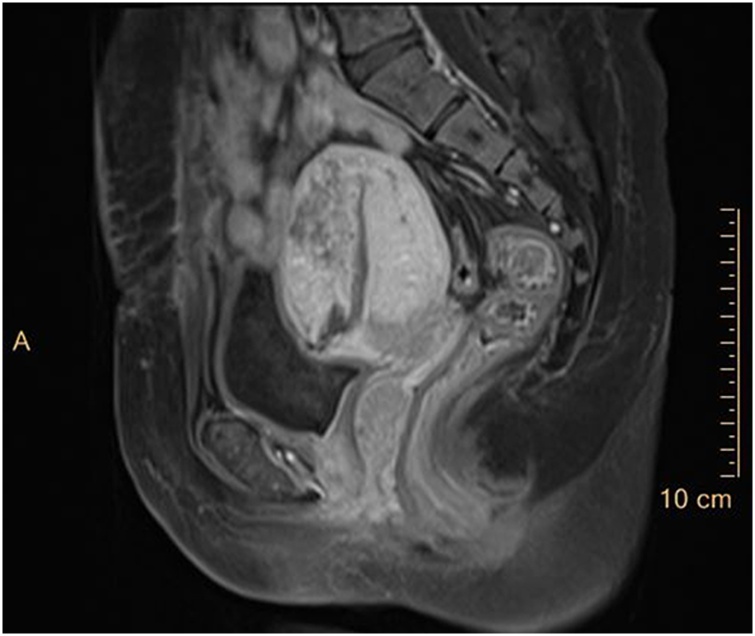
MRI Sagittal (white arrow refers to cancer tissue).

**Fig. 3:**
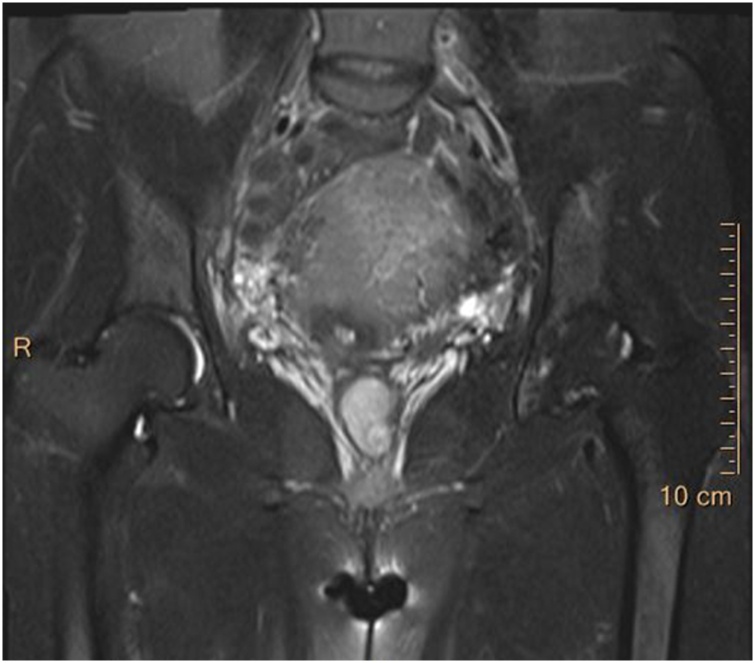
MRI longitudinal section.

Gynaecological examination on admission showed that a hard mass was visible 4 cm above the edge of the hymen in the middle of the left wall of the vagina. The mass was 5 cm × 4 cm × 2 cm, had an uneven surface, a rich supply of blood vessels, thick pedicles, normal mobility, and clear boundaries with the surrounding tissue, and tenderness was positive (Fig. 1). The cervix was squeezed to the right by the mass, and a few old blood clots were seen at the cervix. The anterior position of the uterine body was enlarged to the size of the third month of pregnancy, with clear boundaries, full shape and no tenderness. The attachment did not touch the mass or had no tenderness. No abnormalities were found in the routine blood test, liver and kidney functions, immunity, coagulation function or thyroid gland. Pelvic MRI showed a long T1 (Fig. 2), and slightly longer T2 signals were seen in the vagina with a size of 1.7 cm × 2.1 cm × 3.3 cm (Fig. 3). After strengthening, the signal was significantly strengthened. The rectal wall was not thick. No obvious mass lesions were found. No swollen lymph nodes were found in the pelvis or bilateral groin, and there were no obvious abnormalities in the soft tissue around the pelvis. Vaginal cancer was considered. There were no obvious abnormalities on lung computed tomography (CT) or pelvic ultrasound. Because our department had insufficient experience in vaginal cancer treatment, the patient was discharged on February 19, 2019, and she was recommended to be transferred to Peking Union Medical College Hospital for further treatment.

The pathological diagnosis of vaginal neoplasms was clear cell carcinoma at Peking Union Medical College Hospital. On March 12, 2019, the patient underwent PET-CT, which showed nodular radioactive uptake in the left sidewall of the middle vagina. The left sidewall metabolism increased in the middle of the vagina, and this was consistent with malignant changes. No obvious abnormal metabolic increase was found in the other parts of the vagina. On March 28, 2019, she underwent vaginectomy under general anaesthesia. The postoperative pathology of the vaginal masses was infiltration of adenocarcinoma in the squamous epithelial mucosa. Combined with immunohistochemistry, the lesion was consistent with clear cell carcinoma.

The patient underwent 6 chemotherapy cycles after surgery. The chemotherapy regimen was paclitaxel 300 mg and carboplatin 600 mg, with one course every 21 days. On September 12, 2019, the patient had a CA-125 of 16.2 U/mL after 6 chemotherapy treatments. An ultrasound showed that there were no abnormalities in the uterine appendages. The patient is still being followed up.

## 3 Discussion

Primary vaginal cancer occurs in the vagina and does not affect the cervix or vulva [3]. In 1997, a case of primary vaginal adenocarcinoma was reported by Riva et al. for the first time, and its incidence was rare [4,5]. Approximately 5% of primary vaginal cancers are adenocarcinomas. Subtypes of vaginal adenocarcinoma include clear cell carcinoma, endometrioid carcinoma, and serous and mucinous carcinoma [6]. The cause of vaginal adenocarcinoma is not yet confirmed, but it is often related to women who used diethylstilbestrol (DES) during early pregnancy [7]. It is unclear whether the aetiology of vaginal clear cell carcinoma is related to exposure to DES, and this patient denied a history of exposure to DES.

If a vaginal mass is found during pregnancy, the nature of the mass, its impact on the foetus and pregnancy outcomes must be clarified first, and effective treatment is needed. Because of the rarity of pregnancy with vaginal adenocarcinoma, there are no clinical guidelines to guide treatment. Most treatment options refer to the guidelines for pregnancy with cervical cancer. The patient was at the 29th week of gestation when the vaginal mass was found, but no clear diagnosis of vaginal mass biopsy was made. Vaginal bleeding occurred again at the 30th week of gestation, and the biopsy showed vaginal adenocarcinoma. Because of the high expectation of this pregnancy and the early stage of vaginal cancer, she chose expectant treatment, in which the growth of the tumour was closely observed and examined at the time of delivery. At 37 + 5 weeks of pregnancy, she underwent a caesarean section to deliver a healthy baby girl, followed by further treatment of vaginal cancer. From the results of postoperative follow-up analysis of patients, the timing of choosing to deliver her baby at full term did not have many effects on the treatment of vaginal cancer. However, as the patient chose to deliver her baby at full term, there might be a risk of vaginal cancer metastasis, and the risk of malignant tumours to the foetus is unknown. The patient's treatment plan was vaginectomy and postoperative chemotherapy. Considering that the patient's vaginal cancer stage was stage I and she still had fertility requirements, the scope of surgery had not been expanded. Because she was a young woman, radiotherapy could cause vaginal contracture, radiation cystitis and so on, and these would seriously affect her sexual quality of life in later years, so no radiotherapy was performed.

## 4 Conclusion

The treatment plan of pregnancy with vaginal adenocarcinoma should be individualized. The treatment plan should be determined according to comprehensive factors, such as the gestational week, the stage of the vaginal carcinoma, the expectations and outcome of this pregnancy and the treatment prognosis.

## Declaration of Competing Interest

No conflict of interest.

## Funding

No funding for our research.

## Ethical approval

Not required for case reports at our hospital. Single case reports are exempt from ethical approval in our institution.

## Consent

Written informed consent was obtained from the patient for publication of this case report and accompanying images. A copy of the written consent is available for review by the Editor-in-Chief of this journal on request.

## Author contribution

**Youjiang Xu**: study concept or design, data collection, writing the paper.

**Congqing Li**: study concept or design, data collection.

**Bing Wei**: study concept or design.

**Wenyan Wang**: study concept or design, data analysis, writing the paper.

## Registration of research studies

NA.

## Guarantor

Dr.Wenyan Wang.

## Provenance and peer review

Not commissioned, externally peer-reviewed.
